# Innovations in maternal and child health: case studies from Uganda

**DOI:** 10.1186/s40249-020-00651-0

**Published:** 2020-04-16

**Authors:** Phyllis Awor, Maxencia Nabiryo, Lenore Manderson

**Affiliations:** 1 Makerere University College of Health Sciences, School of Public Health, Kampala, Uganda; 2 School of Public Health, University of the Witwatersrand, Johannesburg, South Africa; 3 School of Social Sciences, Monash University, Melbourne, Australia; 4 Institute at Brown for Environment and Society, Brown University, Providence, RI USA

**Keywords:** Community-based solutions, Maternal and child health, Social innovations, Social innovations in health, Innovations in maternal and child health, Case study research, Uganda

## Abstract

**Background:**

Nearly 300 children and 20 mothers die from preventable causes daily, in Uganda. Communities often identify and introduce pragmatic and lasting solutions to such challenging health problems. However, little is known of these solutions beyond their immediate surroundings. If local and pragmatic innovations were scaled-up, they could contribute to better health outcomes for larger populations. In 2017 an open call was made for local examples of community-based solutions that contribute to improving maternal and child health in Uganda. In this article, we describe three top innovative community-based solutions and their contributions to maternal health.

**Main text:**

In this study, all innovations were implemented by non-government entities. Two case studies highlight the importance of bringing reproductive health and maternal delivery services closer to populations, through providing accessible shelters and maternity waiting homes in isolated areas. The third case study focuses on bringing obstetric imaging services to lower level rural health facilities, which usually do not provide this service, through task-shifting certain sonography services to midwives. Various health system and policy relevant lessons are highlighted.

**Conclusions:**

The described case studies show how delays in access to health care by pregnant women in rural communities can be systematically removed, to improve pregnancy and delivery outcomes. Emphasis should be put on identification, capacity building and research to support the scale up of these community-based health solutions.

## Background

Every day, about 300 neonates and infants and 20 mothers die from preventable causes in Uganda [1]. Most of these deaths occur during delivery and within the first month of life. These deaths are mainly caused by complications to the mother and child in labour and during delivery, and in association with infectious diseases of poverty including malaria, pneumonia, sepsis and HIV/AIDS [2]. These statistics have remained almost the same over the past 10 years, while the Ugandan government (like others in low income countries) is grappling with low human resources for health, lack of medicines, equipment and diagnostics, weak governance, and limited funding for health [3].

In Uganda, maternal mortality is mainly attributed to the “three delays”: delay in making the decision to seek care; delay in reaching a health facility in time; and delay in receiving adequate treatment [4]. The first delay is attributed to the failure of the mother, her family, or the community to recognize a life-threatening condition; in this context, lack of awareness of pregnancy-related health risks is a major reason for the low uptake of maternal health services [5]. The second delay is associated with delays in reaching a health centre, due to road conditions, lack of or cost of transportation, or location of the facility: over 40% of rural women in Uganda report distance-related barriers to accessing healthcare [6]. The third delay occurs at the facility where, upon arrival, women receive inadequate care or ineffective treatment because most health facilities in Uganda, especially in rural areas, persistently lack the necessary medicines and equipment to care for mothers during pregnancy and at the time of and after delivery [7]. The ‘three delays’ model reveals the complexity of maternal health challenges. To tackle these issues, there is need for multi-disciplinary and inclusive approaches that engage various stakeholders, including community members, in solving these problems [8].

Communities often identify and introduce pragmatic and lasting solutions to challenging health problems. Little is known of these solutions beyond their immediate surroundings, but if some of these were scaled-up, they could contribute to better health outcomes for larger populations. In this article, we focus on community-based solutions for maternal health in Uganda.

## Main text

### Study design

The three case studies described in this article were identified through a six-week crowdsourcing call, in May and June 2017, which invited individuals and community organizations to share their community-based solutions to improve maternal and child health in Uganda. The call was launched through newspaper advertisements in the five main local languages in Uganda and through multiple seminars at Makerere University and with the Ministry of Health technical working groups on maternal and child health, e-health and monitoring and evaluation and operational research. The call was further disseminated through different online platforms, print media, and radio advertisements.

Twenty nine nominations were received from diverse implementers across the country. The submitted nominations were within the following categories: improving access to delivery care, for example, by providing maternal waiting homes; phone apps for pregnancy information and for sexual and gender-based violence reporting; improving neonatal care; ultra sound scanning devices; and creating better social and economic opportunities for disadvantaged women and children. Twenty one nominations were eligible and these were reviewed by an external independent panel of judges that included experts from academia, non-governmental organizations and the Ministry of Health. Five top solutions were selected for further case study research.

### Data collection

To better understand the successful social innovations in health, we investigated for novel processes, products, policies, market mechanisms, and practices addressing the health challenges. A descriptive and explorative case study research approach was utilized to understand the selected projects better and to explore the role of social innovation in improving the lives of women and children in Uganda. Further, exploration of cross-case themes that have transferable properties within and between different contexts was undertaken.

Data collection followed the case study methodology as proposed by Yin and Eisenhardt [9] [10] This approach allows for an in-depth systematic exploration of a phenomenon via the collection and analysis of multiple forms of data. Yin proposed the use of six sources of evidence as a way to achieve construct validity in case study research. These include documentation, archival records, interviews, direct observations, participant observations, and physical artefacts [9]. The various forms of data enable an enriched, multi-dimensional layout of the phenomenon of query and supports construct validity.

In this research, data was both qualitative (in depth interviews, observations) and quantitative (evaluation data on the impact of the solution and existing disease and systems indicators on the local health context). Field visits were conducted, and implementers and beneficiaries of the solutions were interviewed. The interviews were recorded and transcribed, and supplementary information was received from the organizations’ records, including reports. This triangulation of multiple forms of qualitative and quantitative data enabled the research team to examine certain aspects in depth, to compare different forms of data around the same aspects, and to constitute or support the coding of a concept using multiple forms of data. Traingulation was also useful for quality control. The collected information was analysed to generate case study reports that reflect the innovative components of each case study and the key health system recommendations for policy makers and implementers.

To support the construction of the social innovation case, data collected through different methods was triangulated as per Table 1 below.

**Table 1:** Case study data collection and data sources

Social innovation case data		
Unit of analyses	Data collection method	Data sources
Context	• Literature Review • In depth interviews • Participant observations	• Peer-reviewed and grey literature • Senior management • Immediate environment, organisation, community
Inventing/ Implementing Actor	• In depth Interview	• Inventor/ Founder of the organisation / CEO
Solution	• Documentation • In depth Interviews • Participant observation	• Grey or published reports on the impact of the solution/ organisation • Founder, CEO, Employees, Beneficiaries • Employees delivering the solution
Organisation	• Documentation • In depth Interviews • Participant observation	• Annual reports of organisation • Founder, CEO, Employees • Organisation operations and daily activities

*CEO* Chief executive officer.

### Case studies

**Fig. 1:**
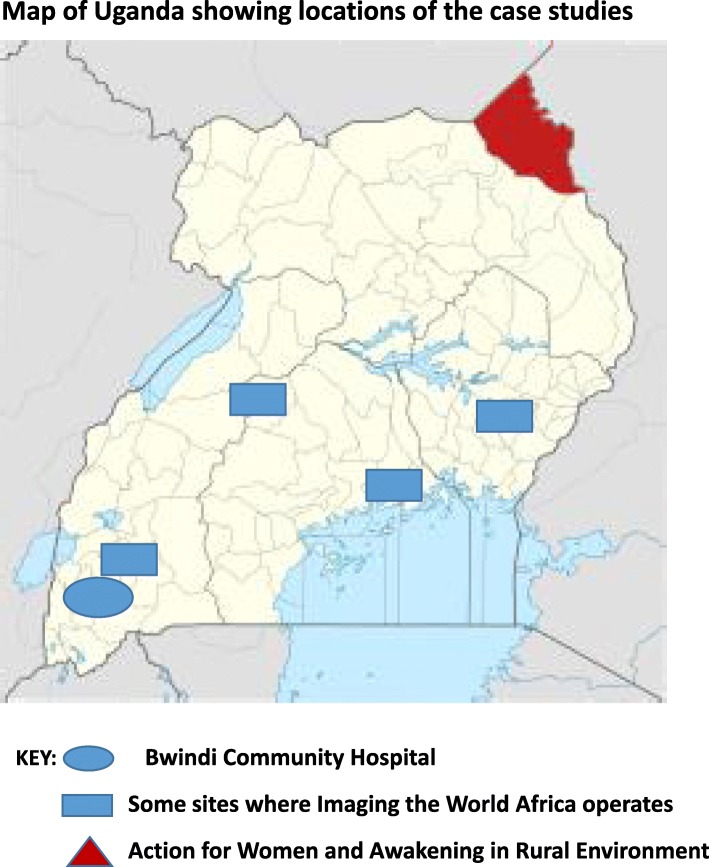
Map of Uganda showing locations of the case studies

Below, we describe three case studies of social innovation in maternal and child health, and provide health system and policy relevant recommendations. Two case studies demonstrate the importance of bringing reproductive health and maternal delivery services closer to recipient populations, through providing accessible shelters and maternity waiting homes in isolated areas. The third case study focuses on bringing obstetric imaging services to lower level rural health facilities, which usually do not provide this service. Figure 1 shows the location of the case studies in Uganda.

### Case 1: mothers’ waiting hostel at Bwindi community hospital

Bwindi Community Hospital (BCH) is a private not-for-profit health facility in South Western Uganda, that has sought to address some of the delays in women’s access to health care by providing a maternity waiting home for pregnant women from remote and hard-to-reach areas for about 1 month prior to expected date of delivery. BCH began as an outreach clinic without fixed facilities — it literally operated under a tree — but it has expanded to a 112-bed hospital which provides health care and health education to the surrounding population.

The hospital serves over 100 000 people, including the Batwa pygmies who lived in the Bwindi forest, and were evicted when the area was made a national park in 1991. The Batwa have been subject to systematic structural violence, with extremely poor health as a result of poverty and displacement. The hospital initially aimed to serve the Batwa, but then expanded to provide health care for other people also in the surrounding sub-counties of Kayonza, Kanyantorogo and Mpungu. The terrain is mountainous and settlements isolated; in consequence, women often walk for approximately 8 h to reach a health care centre [11].

#### The hostel

The waiting hostel was established in 2008 within the BCH to provide pregnant women with a place to stay prior to delivery, so that they did not have to endure long journeys through difficult terrain when they were in labour. By its location within the hospital, the waiting hostel ensured that pregnant women would have access to a skilled birth attendant at delivery. It also ensures that women who are HIV infected are enrolled onto the prevention of mother to child transmission (PMTCT) program, to protect their children from infection. Women are required to make a one-time payment of United States Dollars (USD) 1.5 for the duration of their stay in the hostel. BCH leverages funding from other hospital programs and existing structures, such as sexual and reproductive health services and the Community Based Health Insurance Scheme (CBHI). These services have now been in operation for 10 years.

BCH utilizes existing hospital staff to take care of the women in the waiting hostel. A full time nurse checks each day women’s general condition and vital signs (blood pressure, fetal heart rate etc.). In case of emergency, the fully equipped hospital operating theatre is available and a full time obstetrician is on duty. At the hostel, women prepare their own meals and contribute to cleaning. They also receive basic health education, including on how to prepare nutritious meals for their infants and young children. First time mothers are also engaged in peer learning on how to care for a new-born. The nurses and midwives also conduct sexual health sessions on child spacing, the advantage of small families, and family planning methods, so that women make an informed choice about contraceptive use.

#### The community health worker outreach program

BCH has a community health outreach department with three community health nurses, who work with 502 community health workers in 101 villages to conduct health promotion activities and identify women with high risk pregnancies. Women in the high-risk category as per the WHO definition are especially encouraged to stay at the hostel a few weeks before their expected date of delivery.

#### Impact on health care delivery

From July 2006 to 2012, on average 106 deliveries occurred monthly and an estimated 30% of the mothers utilized the hostel. In 2014, there was a 10.5% increase in women’s utilization of the mothers’ waiting hostel by women from distant sub-counties; and a fourfold increase in the utilization of delivery services at BCH. By 2017, the hospital was delivering an average of 150 babies monthly, and approximately 45–60% of the women utilized the waiting hostel. Thus increasing numbers of women marginalized by location have been accessing the hostel, the antenatal care it provides, and the PMTCT program. In total, following the launch of the health insurance scheme March 2010, there has been a consistent increase in outpatient attendance, inpatient admissions, and deliveries at BCH. Further, about 150 children receive immunization services weekly and all new-born babies received. Bacille Calmette-Guerinand polio vaccines on the maternity ward.

The idea of a maternity waiting hostel is not new in African or other settings. Global guidance on waiting homes in hard-to-reach areas exit, and many countries have related policies [12]. However, in Uganda, there are no publicly run maternity waiting homes. Over 30% of women in rural areas deliver at home, because of continuing barriers to seeking, reaching and receiving quality maternal health care [13]. Distance to a health facility, limited transport services and the direct and indirect costs of travel all influence women’s delivery location, with women living the farthest away from facilities most likely to deliver at home [13–15]. Maternity waiting homes like this one in BCH can contribute to increased access to skilled birth attendants, timely interventions, and better delivery outcomes.

### Case 2: imaging the world, Africa

Due to low income and lack of advanced medical imaging technology, rural women living in remote and under-served areas are unable to access diagnostic imaging, and so have difficulty in receiving timely diagnosis of pregnancy complications. This increases the risk of severe morbidity and mortality among pregnant women. Imaging the World Africa (ITWA) is a Ugandan-registered NGO which focuses on incorporating low-cost ultrasound services into remote health care facilities which routinely do not provide this service, which lack the standard infrastructure required of imaging systems, and where there is a shortage of radiologists. ITWA integrates technology, training and community participation to bring medical proficiency and high-quality imaging services to the population [16].

#### The imaging the world model

The ultrasound program was originally introduced in 2010 to identify high-risk pregnancies in one health facility in eastern Uganda, and expanded to six other districts and 11 facilities by 2016. The model incorporates point of care ultrasound imaging devices, task shifting, training and innovative real-time external radiological expert reviews, using telemedicine services. It combines these services with community awareness and pragmatic funding models that promote self-sufficiency. ITWA provides the program by training nurses and midwives at remote health centres to perform basic ultrasound scans. ITWA developed software to compress and transmit full ultrasound images via the internet to an offsite team of participating radiologists, both in Uganda and abroad, for real-time interpretation, enabling them to review the images, provide a diagnosis, and relay the results back to the transmitting centre.

#### Task-shifting training program

ITWA equips nurses and midwives with the skills and knowledge to conduct obstetric ultrasound scans. They developed a 6 to 8 week certified training program for non-specialist health workers located in rural areas, delivered at the Ernest Cook Ultrasound Research and Education Institute (ECUREI), a private for-profit sonography training centre located in Kampala. Selected midwives or nurses with an expressed interest in sonography undertake practical and theoretical training on how to conduct abdominal sweeps and transmit the images for interpretation. Once health professionals have successfully completed the training course, they are awarded a certificate of completion and ITWA then provides the health facilities in which they are based with ultrasound machines to perform scans.

#### E-health/telemedicine ultrasound radiology service

ITWA developed software (utilizing Digital Imaging and Communications in Medicine) that compresses and transmits full ultrasound images via the internet. During ultrasonography, the probe is passed across the abdomen of the pregnant woman in a series of six prescribed sweeps using a low-frequency transducer, so acquiring a series of static images. These images are de-identified and stored locally on a computer before being compressed and transmitted digitally via an internet connection. They can then be immediately viewed by participating radiologists, the majority of whom are local Ugandan radiologists who volunteer to interpret the scans. An abbreviated report of the findings is sent via SMS to the nurse/midwife’s cell phone, and a full report is sent by email, usually within an hour. In order for this to happen, there must be a laptop, a cell-phone, internet connection, and an ultrasound machine at the point-of-care.

#### Impact on health care delivery

ITWA has rolled out ultrasound services in 11 rural health facilities in Uganda and has trained 150 health workers to perform obstetric ultrasound. Since 2010, 200 000 ultrasound scans have been conducted, with each scan generating data to aid decision making. ITWA maintain that obstetric ultrasound results have helped change the management in 23% of pregnancies with complications. The others did not require imaging for decision making.

The availability of ultrasound scans has allowed pregnant women to receive timely care at the appropriate level of health facility, thereby reducing unnecessary delays and complications of delivery. This has led to an increase in the number of women seeking antenatal care, increased male involvement in ANC services and attendance, because of their interest in seeing an image of the unborn child, and improved birth planning.

Ultrasound sonography has been extended to include echocardiography through a cardiac ultrasound pilot program, with radiologists in the US usually viewing and supporting the interpretation of these images. The pilot program identified 58 pregnant women with heart disease, who were monitored and treated at the clinic close to home. Seven women were monitored for specialized delivery, and one had her first baby after multiple late pregnancy fetal deaths [16]. The US-based radiologists also provide support in interpreting other complex images, such as those taken to determine breast cancer.

### Case 3: action for women and awakening in rural environment (AWARE-Uganda)

AWARE-Uganda is a non-governmental organization operating in three districts of Karamoja region in northeast Uganda: Kaabong, Kotido and Abim districts. Karamoja is the least developed region in the country, with low levels of employment, high levels of illiteracy, food insecurity, poverty and poor health care services, intimate partner violence, and a history of armed conflict, abduction and war-related gender-based violence [17]. The consequences of these challenges, coupled with unfavourable attitudes towards women’s education and community beliefs in the value of early marriage for wealth, have caused great suffering to women and girls in the area [18, 19].

#### The AWARE holistic approach to women’s health and empowerment

AWARE Uganda was established in 1989 by a group of local women in Kaabong district with the aim of advancing the social, cultural and economic status of women in the region [20]. AWARE utilizes a holistic approach to address development issues through women’s empowerment and engagement to improve their own and others’ livelihoods in their community. AWARE provides supportive conditions for women to engage in small business enterprises and agricultural practices, and to increase their roles in leadership and decision making. Women are also sensitized about their rights.

With the establishment of a maternity waiting house, the organization has also improved access to maternal and child health care services, bringing pregnant women closer to Kaabong hospital. As a result, maternal and perinatal morbidity has been reduced.

AWARE-Uganda has engaged and empowered over 5000 women in its activities, including the delivery of an integrated package of services to address the health, economic and social needs of women. Most activities at AWARE are offered by local volunteers, often previous beneficiaries, contributing to the sustainability of the program. Working with men to address negative gender dynamics and to change beliefs around the value of women has been critical, illustrating how empowering and engaging with vulnerable groups and their communities is an effective approach to creating social change.

#### Impact on women’s health

AWARE has conducted community sensitization and capacity building on gender-based violence and intimate partner violence to police officers, health workers, elders, district leaders, and in schools, where child rights clubs have been established in Kaabong district. Community members, including children, are also sensitized on all forms of discrimination against women and human rights, case handling, and reporting procedures. Over 50 girls have been rescued from various forms of violence including gender-based violence and forced marriages, and have received counselling from AWARE staff who also link them to treatment at Kaabong hospital.

In 2016, AWARE Uganda conducted 28 training workshops for ten women’s groups on the use of modern farming methods, including the use of ox ploughs, crop spacing, and making and using composite manure to improve soil quality and crop yields. These skills were shared with over 370 households. AWARE purchased 25 ploughs and 25 ox chains, and 550 hoes, pangas and axes to assist women in agriculture. About 200 women from four communities were involved in chilli and honey production, improving their livelihood and those of their families.

AWARE also runs a mother’s waiting home in the semi-arid Karamoja region. The 20-bed maternal waiting home at the AWARE centre was established in 2010 and is the only one of its kind in the area. Since this date to time of writing (2019), over 500 women have received services at this facility per annum, including antenatal case, clinical monitoring when the pregnant women is resident at the home, and skilled delivery care; many more receive health education information. About 1000 people have utilized family planning services provided at AWARE.

With support from partners, AWARE distributed 12 040 home health care kits, including condoms, to community members in Kaabong district. AWARE registered and trained 32 Village Health Teams (VHTs) to operate in five sub-counties, with VHTs following up on those who need care at household or community level.

Leveraging community social capital as a resource for this organization was pivotal. The founders did not wait for funding opportunities to start organizing women, but rather, drew on women’s ideas, energy and time. Women asked for land from the district government and were granted this. They then bought and planted 150 fruit tree seedlings, and this marked the start of their activities.

Utilizing volunteers and beneficiaries was key to sustaining AWARE’s efforts, and it has operated for 30 years in these rural areas. Women have become empowered to support other women in similar situations. AWARE believes in working with partners to strengthen and advance work, and in this context, the police and Kaabong Main Hospital work together to support the organization in addressing gender-based violence, receiving and attending to referrals from the organization. One major challenge that AWARE had was to overcome negative attitudes towards women, and to change men’s mind set, AWARE started involving men in activities while working to empower women. AWARE has therefore shown that it is possible to overcome discriminatory cultural perceptions and practices through committed long-term involvement.

## Discussion

These three cases provide innovative and pragmatic solutions to the three delays in access to health care, which are known to significantly contribute to maternal mortality in Uganda. When pregnant women in remote and hard to reach locations access and utilize maternal waiting homes prior to the onset of labour and delivery, this immediately removes the problem of recognition of danger signs in pregnancy, as well as that of delayed health care decision making and lack of access to a skilled birth attendant. In addition, taking ultrasound imaging closer to pregnant women, also directly contributes to reductions in all the three delays. This is through early recognition of high risk pregnancies like multiple pregnancies and placenta previa and decision making related to birth planning and delivery.

### Key health system lessons

Based on these case studies, three key health system lessons emerge:

The first is that while maternity waiting homes for high-risk pregnant women in remote areas are recommended in national and global health policies, they are almost non-existent in Uganda and other low income settings. Maternity waiting homes can contribute to increasing institutional deliveries, reducing obstetric delays and improving maternal and perinatal health outcomes in remote areas. In hard to reach areas, maternity waiting homes may contribute to reducing the high maternal deaths. As shown above, the waiting home can also provide opportunities for health education for mothers to improve the wellbeing of their new born children and families. For stronger effect, CHW outreach programs can contribute to identifying and getting women into hospital in remote and inaccessible areas.

The second health system lesson relates to the important role of shifting some acceptable health care roles from higher qualified to less qualified health workers (task shifting). The majority of community-based innovations identified within the SIHI involved some task-shifting activities. As we have illustrated for ITWA, task shifting can create an effective way to deliver ultrasound services to low resource settings. Trained midwives can conduct the ultrasound scan, reducing the cost of hiring a sonographer in low resource and remote settings. In addition, the integration of telemedicine for the interpretation of ultrasound scans is feasible and provides an opportunity to improve the quality of care to patients.

Thirdly, in order to contribute to effective social change for women experiencing discrimination and violence, full community and multi-sectoral action is necessary, including men’s participation in women’s empowerment and increased decision making. The bottom up approach utilised by AWARE is important for effective change. AWARE works to ensure that all community members (men and women) have skills to improve their livelihoods and to support gender equality. Past program beneficiaries, for example, women and girls who experienced GBV, can become active providers of services to new beneficiaries, sensitizing them about gender-based violence and contributing to sustainability.

### Principles of social innovation

All these cases also demonstrate the principles of social innovation [21, 22]. These are: strong community participation; multi-stakeholder engagement; addressing gaps in health and wellbeing (needs-based); and contribution to transformation in the health and lives of beneficiaries. Additional characteristics of the three case studies are that they are complementary to public health care provision and they focus on improving access to health care (affordability of services, bringing services closer to the people, and utilization of task-shifting mechanisms).

Affordability is a key component of these social innovation solutions, as services must be provided at an affordable price, so that communities can access them consistently, and sustainably. Two of the solutions request a user fee of about USD 1.5, while AWARE provides free services, sustained by the grants it receives.

Finally, availability of health services and geographical access are key components, which are addressed in these case studies through the utilization of lay community health workers to provide health services and through task shifting and training midwives for obstetric imaging service provision.

## Conclusions

The ability of communities to identify and implement practical solutions to health care challenges in low income settings needs to be recognised and embraced. The described case studies show how delays in access to health care by pregnant women in rural communities can be systematically removed, to improve pregnancy and delivery outcomes. Stronger emphasis should be put on identification, capacity building and research, in order to support the scale up of these community-based health solutions.

## Data Availability

Original case studies are available online at https://socialinnovationinhealth.org/uganda/

## Abbreviations

ANC: Antenatal care
AWARE: Action for Women and Awakening in Rural Environment
BCG: Bacille Calmette-Guerín
BCH: Bwindi Community Hospital
CBHI: Community Based Health Insurance Scheme
CEO: Chief executive officer
CHW: Community health worker
ECUREI: Ernest Cook Ultrasound Research and Education Institute
GBV: Gender Based Violence
ITWA: Imaging the World Africa
MWH: Mothers’ Waiting Hostel
NGO: Non-governmental organization
SIHI: Social Innovation in Health Initiative
TDR: Special Programme for Research and Training in Tropical Diseases
USD: United States dollar
VHT: Village health team
